# Metabolic Health Has Greater Impact on Diabetes than Simple Overweight/Obesity in Mexican Americans

**DOI:** 10.1155/2016/4094876

**Published:** 2016-01-10

**Authors:** Shenghui Wu, Susan P. Fisher-Hoch, Belinda Reninger, Kristina Vatcheva, Joseph B. McCormick

**Affiliations:** ^1^Department of Epidemiology & Biostatistics, University of Texas Health Science Center at San Antonio, Laredo Campus, Laredo, TX 78045, USA; ^2^Division of Epidemiology, School of Public Health, University of Texas Health Science Center at Houston, Brownsville Campus, Brownsville, TX 78520, USA

## Abstract

*Purpose*. To compare the risk for diabetes in each of 4 categories of metabolic health and BMI. *Methods*. Participants were drawn from the Cameron County Hispanic Cohort, a randomly selected Mexican American cohort in Texas on the US-Mexico border. Subjects were divided into 4 phenotypes according to metabolic health and BMI: metabolically healthy normal weight, metabolically healthy overweight/obese, metabolically unhealthy normal weight, and metabolically unhealthy overweight/obese. Metabolic health was defined as having less than 2 metabolic abnormalities. Overweight/obese status was assessed by BMI higher than 25 kg/m^2^. Diabetes was defined by the 2010 ADA definition or by being on a diabetic medication. *Results*. The odds ratio for diabetes risk was 2.25 in the metabolically healthy overweight/obese phenotype (95% CI 1.34, 3.79), 3.78 (1.57, 9.09) in the metabolically unhealthy normal weight phenotype, and 5.39 (3.16, 9.20) in metabolically unhealthy overweight/obese phenotype after adjusting for confounding factors compared with the metabolically healthy normal weight phenotype. *Conclusions*. Metabolic health had a greater effect on the increased risk for diabetes than overweight/obesity. Greater focus on metabolic health might be a more effective target for prevention and control of diabetes than emphasis on weight loss alone.

## 1. Introduction

The proportion of overweight/obese adults in the US increased between 1980 and 2013 from 28.8% to 36.9% in men and from 29.8% to 38.0% in women [1]. Overweight/obesity increases the risk for type 2 diabetes mellitus [2, 3]. However, being metabolically unhealthy also increases the risk for type 2 diabetes [4–6]. Though these findings suggest that the risk for type 2 diabetes associated with overweight/obesity is influenced by the coexistence of metabolic abnormalities, the independent impact of these two conditions is unclear. Metabolically unhealthy normal weight (MUHNW) subjects are individuals with normal weight but with metabolic abnormalities [7, 8], but the phenotype is ill-defined. Among normal weight individuals aged 20 years or older in the Third National Health and Nutrition Examination Survey, 4.6% of men and 6.2% of women had three or more metabolic abnormalities [9]. Because MUHNW subjects are not overweight or obese, they may not be aware of their risks and may be missed and therefore may not benefit from adequate prevention. Nevertheless, MUHNW carries a significant risk for cardiovascular diseases [10, 11] and mortality [12]. The findings from several studies have shown the increased risk of diabetes in MUHNW individuals [4–6] compared with metabolically healthy normal weight (MHNW) individuals in different ethnicities, but only one study was conducted in Mexican Americans [6].

There is no consensus on the risk presented by the metabolically healthy overweight/obese (MHOW) phenotype [4, 5]. Evidence regarding the risk of diabetes associated with the MHOW phenotype is also uncertain. It has been reported that MHOW individuals may be more likely to develop incident diabetes compared with normal weight individuals [4–6]; however data from Kangbuk Samsung Health Study of more than 6 thousand individuals did not show a significant association with diabetes [5] and data in Mexican Americans is in any event limited [6]. Although metabolically unhealthy overweight/obese (MUHOW) subjects showed a higher risk of diabetes than MHNW phenotype [4–6, 13], only a few studies compared the diabetes risk between MUHOW, MUHNW, MHOW, and MHNW phenotypes [4–6] and only one was conducted in Mexican Americans [6].

Mexican Americans have higher prevalence of diabetes, overweight/obesity, and metabolic disturbances than non-Hispanic Whites [14–16]. The objective of this study was to compare the risk for diabetes among the 4 phenotypes divided by metabolic health and overweight/obesity status in a randomly selected cohort of Mexican American subjects.

## 2. Materials and Methods

### 2.1. Study Participants

This study was approved by the Committee for the Protection of Human Subjects of the UT Health, Houston and the Institutional Review Board of the University of the Texas Health Science Center, San Antonio. All study participants gave written informed consent. This cross-sectional analysis used data from the Cameron County Hispanic Cohort (CCHC), a homogenous community-dwelling Mexican American ongoing cohort study [17, 18]. Study subjects were recruited from randomly selected blocks according to the 2000 Census as described previously [17, 18]. At the baseline survey conducted between 2003 and 2014, 3,257 participants aged 18 years or older were recruited from their households in predominantly Mexican American cities along the Rio Grande border with Mexico. To reduce the effect of type I diabetes on the results, the participants who had diabetes before 18 years were excluded (*n* = 10).

All subjects responded to a detailed baseline survey of demographic characteristics, lifestyle including diet, physical activity, family, and medical history, and other exposures. Participants were asked to fast for at least 10 hours overnight before a clinic visit at the clinical research unit. Anthropometric measurements, including current weight, height, and circumferences of the waist and hip, were also taken [17, 18].

Weight was measured to the nearest tenth of a kilogram and height to the nearest tenth of a centimeter. Body mass index (BMI) was calculated as weight in kilograms divided by height squared in meters (kg/m^2^). Waist circumference (WC) was measured at the level of the umbilicus and hip circumference (HC) at the level of maximum width of the buttocks with participants in a standing position and breathing normally, to the nearest 0.2 cm. Waist-to-hip ratio (WHR) was calculated as WC divided by HC [17]. Body fat percentage was estimated using the resistance values from the Quantum X bioelectric body composition analyzer with the sex-specific equations from Sun et al. [19]. The average of 3 blood pressure (BP) measurements taken 5 minutes apart were used.

All participants completed a detailed baseline survey that collected information on demographic characteristics, lifestyle and dietary histories, medical history, and other exposures. Physical activity was assessed using the International Physical Activity Questionnaire (IPAQ) short form [20]; reported minutes of physical activity per week were weighted by a metabolic equivalent (MET, multiples of resting energy expenditure) resulting in a physical activity estimate expressed as MET-minutes per week [20]. Physical activity energy expenditure was estimated using standard metabolic equivalent (MET) values [20].

### 2.2. Laboratory Measurements

All participants donated a blood sample at baseline. After collection, samples were placed on ice and centrifuged within 30 minutes of collection. Following processing and aliquoting, all samples were stored at −80°C until laboratory analyses were conducted. Laboratory studies performed included fasting lipid panel, hemoglobin (Hb) A1c, fasting plasma glucose, and fasting serum insulin. Homeostasis model assessment insulin resistance index (HOMA-IR) was calculated as fasting glucose (mg/dL)/18 × fasting insulin (mU/L)/22.5 [21]. High sensitivity C-reactive protein (CRP) levels were measured using Quantikine ELISA kit (R & D Systems, Inc., Minneapolis, USA).

### 2.3. Identification of the Overweight/Obese and Metabolic Health

Participants were categorized as overweight/obese or with normal weight using a BMI cutoff of 25.0 kg/m^2^ [1] and then were further categorized as metabolically healthy or unhealthy. Metabolic health was defined as having <2 of the following metabolic abnormalities: systolic BP (SBP) ≥ 130 mmHg and/or diastolic BP (DBP) ≥ 85 mmHg or on antihypertensive medication; triglyceride ≥ 150 mg/dL; high-density lipoprotein cholesterol < 40 mg/dL in men or <50 mg/dL in women; or HOMA-IR value > 90th percentile [22, 23]. Waist circumference was not included due to its high correlation with BMI [22]. To avoid bias we did not use blood glucose levels nor diabetes medication in the definition of metabolic health so as to compare the risk for diabetes in 4 phenotypes of metabolic health and BMI.

According to the above criteria, participants were divided into four phenotypes:

1. MHNW: metabolically healthy, normal weight: BMI < 25 kg/m^2^ and <2 metabolic risk factor;
2. MHOW: metabolically healthy, overweight/obese: BMI ≥ 25 kg/m^2^ and <2 metabolic risk factor;
3. MUHNW: metabolically unhealthy, normal weight: BMI < 25 kg/m^2^ and ≥2 metabolic risk factor;
4. MUHOW: metabolically unhealthy, overweight/obese: BMI ≥ 25 kg/m^2^ and ≥2 metabolic risk factor.

### 2.4. Identification of Diabetes

Diabetes was identified by the 2010 definition of diabetes of the American Diabetes Association [24] or the participants reporting being told by a health care provider that they had diabetes or if they were taking hypoglycemic medication.

### 2.5. Statistical Analysis

Descriptive results and the models reported in this paper were adjusted for the probability of sampling using weights taking into consideration clustering effects arising from the census block and household [17]. Log-transformation was conducted to normalize the distribution of the biomarkers studied as appropriate. Survey-weighted linear regression was used to obtain the *t*-test statistics to compare phenotypes and to be used for multiple pairwise mean comparisons for continuous data. Survey-weighted chi-square test was used to obtain Rao-Scott *F* adjusted chi-square statistic to compare phenotypes for categorical data. Survey-weighted logistic regression modeling was performed to estimate the odds ratios (ORs) for diabetes risk and their 95% confidence intervals (CIs) by the metabolic health and overweight/obese phenotype phenotypes adjusting for other covariates. Initially, a multivariable survey-weighted logistic regression model was created to identify independent factors associated with diabetes, among variables including the overweight/obese phenotypes, age, gender, education, reported minutes of physical activity per week, servings of fruits and vegetables per day, and alcohol drinking and cigarette smoking status. Variables that were not significant and were not confounders were excluded from the final model. The interaction effects between the independent variables were tested. The analysis involved in physical activity and dietary data was conducted in 2,044 participants because the interview was only administered in these subjects.

To compare the risk of diabetes in different metabolic health and overweight/obese phenotypes, we also used a restricted cubic spline logistic regression analysis [25] to evaluate the risk of diabetes with age (*P* < 0.0001 for all participants) stratified by metabolic health and overweight/obese phenotypes. Knots were placed at the 5th, 50th, and 95th percentiles of the distribution of age at enrollment. We excluded participants whose age at enrollment was below 20 or above 70 from the restricted cubic spline model to minimize the influence of outliers.

Sensitivity analyses were performed including fasting glucose > 100 mg/dL or on hypoglycemic medication as a component for the definition of metabolic health (metabolically healthy, 0 metabolic abnormalities; metabolically unhealthy, ≥2 metabolic abnormalities) [22, 23]. Participants were categorized as overweight (25–29.9 kg/m^2^) or obese (≥30 kg/m^2^) using BMI cutoffs of 25.0 and 30 kg/m^2^ [1] and separate analyses by overweight and obesity were also conducted. Statistical analyses were carried out by using SAS version 9.3 (SAS Institute, Cary, NC). All statistical tests were based on two-sided probability.

## 3. Results

At the time of this study a total of 3,257 individuals were enrolled in the CCHC, 2,893 participants from Brownsville and 242 participants from Harlingen (Lower Rio Grande Valley) and 138 participants from Laredo (Webb County), Texas. Among 3,247 remaining participants after excluding 10 subjects who developed diabetes before 18 years of age (to minimize potential type I diabetes), 475 subjects (14.6%) were classified as MHNW, 1,594 (49.1%) as MHOW, 72 (2.2%) as MUHNW, and 1,106 (34.1%) as MUHOW (Table 1). Mean age of this subset was 46 years; 34% were male. A total of 36.3% (*n* = 1,178) were classified as metabolically unhealthy. Detailed characteristics by overweight, obesity, and metabolic health were shown in Supplemental Table  1 (see Supplementary Material available online at http://dx.doi.org/10.1155/2016/4094876).

Metabolically unhealthy phenotypes showed significantly elevated mean values of total cholesterol, triglycerides, high-density lipid cholesterol (HDLC), fasting glucose and insulin, HOMA IR, HbA1c, CRP, and blood pressure compared with metabolically healthy phenotypes. They were more likely to be older, cigarette smokers and unemployed, less well educated, and less likely to meet the recommended guidelines for physical activity of more than 600 MET-minutes/week. They had lower household income but more frequent family history of diabetes (all* P*s < 0.05). There was no difference in gender between metabolically unhealthy and healthy phenotypes (Table 1).

Overweight/obese phenotypes were also more likely to be older, less well educated, and cigarette smoking, had lower incomes but more frequent family history of diabetes, and showed significantly elevated mean values of total cholesterol, triglycerides, HDLC, fasting glucose and insulin, HOMA IR, HbA1c, CRP, blood pressure, BMI, WC, WHR, and body fat percentage compared with normal weight phenotypes (all* P*s < 0.05). There was no difference in gender, employment status, and physical activity between overweight/obese and normal weight phenotypes (Table 1).

Seventy-two participants of the cohort (2.2%) were classified as metabolically unhealthy, normal weight. Compared with other three phenotypes, MUHNW subjects were more likely to be older, unemployed, and cigarette smoking, were least likely to meet the recommended guidelines for physical activity of more than 600 MET-minutes/week, and had least income and worst mean values in total cholesterol, SBP, and HbA1c (all* P*s < 0.05) (Table 1).

**Figure 1:**
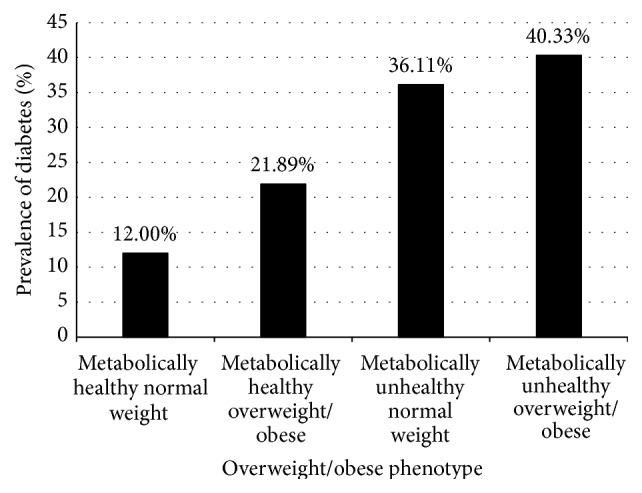
Prevalence of diabetes among Mexican Americans within each overweight/obese phenotype. Black bars indicate prevalence rate. Diabetes prevalence was different by overweight/obese phenotype (*P* < 0.0001).

A total of 878 (27.04%) participants had diabetes (Table 2). Among the four phenotypes the MHNW phenotype had the lowest rates of diabetes (12%) and the MUHOW phenotype had the highest (40.3%) (*P* < 0.0001) (Figure 1). Metabolically unhealthy subjects showed significantly higher diabetes prevalence than metabolically healthy subjects (40.3% versus 19.6%; *P* < 0.0001). Overweight/obese phenotypes showed significantly higher diabetes prevalence than normal weight phenotypes (29.4% versus 15.2%; *P* < 0.0001).

Overweight/obese individuals showed an OR of having diabetes of 2.06 (95% CI: 1.33–3.21) after adjusting for age and metabolic health. Poor metabolic health was positively related to the increased risk of diabetes (OR = 2.46; 95% CI: 1.88–3.21) after adjusting for age and overweight/obesity, suggesting that being metabolically unhealthy carried a higher risk for diabetes than being overweight/obese (2.46 versus 2.06). The risk of diabetes by categories of BMI and metabolic status is shown in Table 2. In a multivariable adjusted logistic regression model with diabetes as the dependent variable, MHOW subjects showed an OR of having diabetes of 2.25 (95% CI 1.34–3.79), MUNW individuals showed an OR of 3.78 (95% CI 1.57–9.09), and MUHOW subjects showed an OR of 5.39 (95% CI 3.16–9.20) after adjusting for confounding factors with the MHNW phenotype as the reference (Table 2). The biggest effect comes from being metabolically unhealthy and normal weight: the adjusted odds ratio for this group compared to the metabolically healthy and normal weight one is 3.8, while the OR for metabolically unhealthy and normal weight compared to metabolically healthy and normal weight is just over 2 (Table 2). The addition of metabolically unhealthy phenotypes to obesity increases the OR for diabetes to over 5-fold (Table 2). The ORs for the risk of diabetes were greater than 1 for the metabolically unhealthy phenotype and were much higher than the ORs for MHNW and MHOW phenotypes: the difference of ORs between MUHNW and MHNW phenotypes was 278%; and the difference of ORs between MUHOW and MHOW phenotypes was 140%, while the difference of ORs between MHOW and MHNW phenotypes was 125%, and the difference of ORs between MUHNW and MUHOW phenotypes was 43%. These comparisons suggested that the risk of diabetes in metabolically unhealthy phenotype was higher than healthy phenotype in any category of BMI, and the metabolic health is more important than simple overweight/obesity. Restricted to the participants who had available data for family history of diabetes (68%), the ORs in each phenotype were not materially changed. When fasting blood glucose > 100 mg/dL or being on hypoglycemic medication was included as a component for the definition of metabolic health in the sensitivity analysis, the correlation was not significant for the MHOW phenotype, the correlation for the MUHNW phenotype remained similar, and the correlation for the MUHOW phenotype became slightly stronger. The sensitivity analysis results further suggested that the metabolic health is more important than simple overweight/obesity. Supplemental Table  2 further showed the risk of diabetes by overweight, obese, and metabolic status. Similar patterns as in Table 2 indicated that the risk of diabetes in metabolically unhealthy phenotype was higher than healthy phenotype, and the metabolic health is more important than simple overweight or obesity.

**Figure 2:**
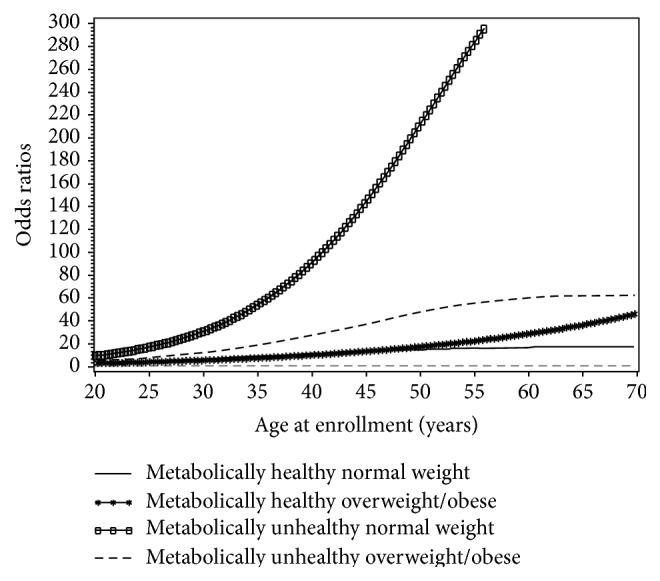
Smoothed plot for odds ratios (ORs) of the diabetes risk according to age at enrollment. Subjects were divided into four phenotypes according to overweight/obese phenotype and metabolic health status. The ORs were estimated by using the restricted cubic spline logistic regression models with knots placed at the 5th, 50th, and 95th percentiles of age at enrollment. The models were adjusted for the probability of sampling using weights taking into consideration clustering effects arising from the same census block and household. The linear correlation between age at enrollment and the risk of diabetes in each phenotype was significant (*P* = 0.02, 0.0001, 0.04, and <0.0001 for phenotypes with metabolically healthy normal weight, metabolically healthy overweight/obesity, metabolically unhealthy normal weight, and metabolically unhealthy overweight/obesity, resp.).

Figure 2 visually depicts the shape of the correlation between age and diabetes risk in four phenotypes after adjusting for potential confounding variables in a restricted cubic spline model. Age was positively and approximately linearly associated with the risk for diabetes in each phenotype (all* P*s < 0.05). Metabolically unhealthy phenotypes had higher ORs than their corresponding counterparts in any category of BMI, and MUHNW phenotype had the highest ORs in the four phenotypes. MHOW subjects had higher ORs than MHNW subjects, and the latter had the lowest ORs in the four phenotypes.

## 4. Discussion

In a Mexican American cohort metabolically unhealthy subjects showed significantly increased risk for diabetes compared with metabolically healthy subjects in any category of BMI. Compared with the metabolically healthy normal weight participants (MHNW), the metabolically unhealthy, regardless of their BMI (MUHNW and MUHOW), and the metabolically healthy obese (MHOW) phenotypes had significantly increased risk of diabetes. MUHNW individuals had a fourfold increased risk and MUHOW individuals had a fivefold increased risk for diabetes compared with the MHNW phenotype. Cubic spline interpolation showed that the risk of diabetes with age was higher in metabolically unhealthy phenotype than metabolically healthy phenotype in any category of BMI. The significance of these observations is that poor metabolic health puts the individual at greater risk of diabetes than obesity alone.

A high proportion of the Mexican Americans in our population are metabolically unhealthy (36.3%) but over half (59.9%) if the definition of metabolic health includes a glucose component. Because we wanted to examine the relationship of diabetes, a disease of glucose metabolism, we excluded that from our criteria for metabolic abnormalities, yet metabolic abnormalities remain the major association with diabetes. Others have shown a high prevalence of metabolically unhealthy Mexican Americans (30%) [6]. In general, Mexican Americans also have high prevalence of diabetes, overweight/obesity, and metabolic abnormalities compared to non-Hispanic Whites [14–16]. Using both logistic regression and cubic spline models, being metabolically unhealthy posed a significantly higher risk for diabetes than being overweight. Similar observations have been made in a prospective cohort study in 6,748 Koreans [5], although the results were not all statistically significant among different overweight/obese and metabolic health phenotypes [5]. Furthermore, we found that metabolically unhealthy overweight/obese individuals were more likely to develop incident diabetes compared with their normal weight counterparts which was consistent with other findings [4–6]. An important element here is the identification of those with metabolic risk factors but who are not obese since they are at high risk for diabetes but less likely to be identified early and provided prevention education. These individuals may well be overlooked in screening programs.

It is not surprising that poor metabolic health puts the individual at a higher risk of diabetes than overweight/obesity alone. In our study, metabolically unhealthy phenotype tended to do less exercise and had lower education and less income level but had increased cigarette smoking compared with metabolically healthy phenotypes. In particular, the MUHNW phenotype showed the lowest proportion of subjects who met minimum recommendations for physical activity of ≥600 MET-minutes/week despite their normal weight, although physical activity was not significant in multivariable analysis for the risk of diabetes. Because physical activity was not correlated with diabetes risk (*P* = 0.18) and metabolic health (*P* = 0.07) in logistic regression models and it was not statistically significant in the multivariable adjusted model (*P* = 0.46), it was not adjusted for in the final model.

Metabolically unhealthy phenotype had higher markers of inflammation which may be the key underlying pathology. Metabolically unhealthy subjects showed significantly higher levels of CRP compared with their metabolically healthy counterparts, consistent with the known role of systemic inflammation in the risk of diabetes. Several conditions that are driven by inflammatory processes are also associated with diabetes, including rheumatoid arthritis, gout, psoriasis, and Crohn's disease, and various anti-inflammatory drugs have been approved or are in late stages of development for the treatment of these conditions [26]. Another important difference between metabolically healthy and unhealthy phenotypes was markedly higher dyslipidemia, measured as hypertriglyceridemia and low HDL-C, fasting glucose, high blood pressure, or insulin resistance observed in the metabolically unhealthy phenotype. These results suggest the importance of lifestyle modification and control of systemic inflammation in maintaining metabolic health and normoglycemia, not the simple reduction in body weight, although physical activity was not significant in multivariable analysis for the risk of diabetes.

Our study found a positive dose-response with approximately linear relationship between age and the risk of diabetes in each phenotype, stratified by overweight/obesity and metabolic health. Metabolically unhealthy phenotypes had higher risk than their corresponding counterparts in any category of BMI, and MUHNW phenotype had the highest ORs in the four phenotypes. To our knowledge, this is the first study to find the risk of diabetes with age higher in metabolically unhealthy phenotypes than in metabolically healthy phenotypes in any category of BMI. The Korean Healthy Twin Study (*n* = 2,687) reported that the risk of diabetes was 4.4-fold higher in MUHNW individuals than in MHNW individuals and 3.3-fold higher in MUHOW subjects than in MHNW subjects [27]. Despite a normal weight identical to the MHNW subjects, MUHNW subjects in our study presented an increased fasting serum insulin and blood glucose, HOMA IR, and HbA1c. This phenomenon may be associated with impaired insulin sensitivity (euglycemic hyperinsulinemic clamp or oral glucose tolerance test) [11, 28]. Although the mechanism is still not clear, at least we are now aware that the MUHNW individuals are a target population in which to identify and to prevent diabetes [27].

Previous studies have suggested that metabolic healthy overweight/obesity was not a benign condition [4–6]. MHOW individuals may be more likely to have diabetes compared with metabolically healthy normal weight peers [4, 6]. Our study found that MHOW subjects had a significant 2.14-fold elevated risk of diabetes (Table 2) and they were more likely to do this before 50 years of age compared with their MHNW counterparts (Figure 1). However, in our sensitivity analysis including fasting glucose as a component for the definition of metabolic health, the risk of diabetes was not significant for the MHOW phenotype. Clearly, the risk of diabetes in MHOW populations needs to be further investigated.

Although a small proportion of the subjects (2.22%) were classified as metabolically unhealthy normal weight phenotype, their risk for diabetes was higher than MHNW and MHOW (Table 2) and they had the highest ORs for the correlation between age and diabetes risk in the four phenotypes (Figure 2). The proportion might be increased if there is no effort to protect this population, because these subjects are not overweight or obese and they may escape detection and therefore not benefit from adequate treatment or prevention measures. Furthermore, the interventions to boost metabolic health involved changes in lifestyles such as diet, exercise, and behavior (smoking and others) which may not be related to overweight/obesity [29]. Therefore, it is very important to make efforts on improving metabolic health in any categories of BMI. The MUHNW phenotype needs to be included within the scope of prevention and control, but should not be ignored.

There are some methodological limitations in our research. The study was cross-sectional in design; thus, only association but not causal relationship may be inferred. Prospective studies are needed to further investigate whether metabolic health is more important than overweight/obesity alone. Our longitudinal data currently being collected will provide that opportunity. We could not completely rule out the possibility of residual confounding due to unmeasured or inadequately measured covariates such as the missing values with some variables.

This study had several strengths. First, this is a general population-based randomly selected Mexican American cohort, thus avoiding bias inherent in studies drawn from clinic populations or other nonrandomly selected populations with established disease or mixed ethnicity. Second, detailed information on a wide range of factors related to diabetes was available, allowing us to get a relatively comprehensive analysis of the affecting factors. Third, cubic spline interpolation was used to compare the dose-response correlation between age and the diabetes risk in different metabolic health and overweight/obese phenotypes and suggested the importance of metabolic health compared to overweight/obesity for the risk of diabetes associated with age. Finally, published studies generally only compared the disease status between obesity (BMI ≥ 30 kg/m^2^) and normal weight phenotypes stratified by the metabolic health status, while the risk of diseases for the phenotype with BMI 25–30 kg/m^2^ was neglected [6, 30]. However, previous studies [2, 3] and our study found that overweight/obese individuals (BMI ≥ 25 kg/m^2^) were at a higher risk of diabetes compared with normal weight individuals.

In conclusion, in our cohort those who had more than two markers indicating unhealthy metabolism had statistically higher prevalence and odds of having diabetes compared to those with healthy metabolism suggesting a higher risk of diabetes adjusting for age, gender or BMI, and overweight/obese. Therefore, being metabolically unhealthy is likely more important for the risk of diabetes than simply being overweight/obese. Efforts need to be focused on improving metabolic health in all categories of BMI. Early lifestyle intervention in these populations is likely to be more effective than simple weight loss.

## Supplementary Material

Similar patterns as in Table 1 showed that metabolically unhealthy phenotypes had significantly elevated mean values of total cholesterol, triglycerides, high density lipid cholesterol (HDLC), fasting glucose and insulin, HOMA IR, HbA1c, CRP, and blood pressure compared with metabolically healthy phenotypes. They were more likely to be older, cigarette smokers and unemployed, less well educated, and less likely to meet the recommended guidelines for physical activity of more than 600 MET-minutes/week. They had lower household income but more frequent family history of diabetes (all Ps < 0.05).

## Figures and Tables

**Table 1:** Cohort demographics and metabolic characteristics stratified by overweight/obese type and metabolic health status: Cameron County Health Cohort Study (2003–2014)^1,2^.

Variable		Metabolically healthy		Metabolically unhealthy		*P* value
	Total	Normal weight	Overweight/obese	Normal weight	Overweight/obese	
	(*n* = 3247)	(*n* = 475, 14.63%)	(*n* = 1594, 49.09%)	(*n* = 72, 2.22%)	(*n* = 1106, 34.06%)	
Categorical variables, *n* (%)						
Men	1108 (34.11)	139 (28.64)	544 (38.09)	25 (33.75)	400 (33.63)	0.054
Employed	1618 (49.83)	242 (50.95)	822 (51.57)	25 (34.72)	529 (47.83)	0.03
Education, below high school	1693 (52.14)	195 (41.05)	813 (51.00)	37 (51.39)	648 (58.59)	<0.0001
Met minimum recommendations for physical activity of ≥ 600 MET-minutes/week	337 (10.38)	48 (10.11)	192 (12.05)	1 (1.39)	96 (8.68)	0.02
Met recommendations of ≥ 5 servings of fruit and vegetables per day	113 (3.48)	9 (1.89)	67 (4.20)	4 (5.56)	33 (2.98)	0.32
Current smokers	495 (15.24)	64 (13.47)	222 (13.93)	14 (19.44)	195 (17.63)	0.02
Ever smokers	984 (30.3)	119 (25.05)	478 (29.99)	21 (29.17)	366 (33.09)	0.0003
Ever alcohol drinkers	1230 (37.88)	186 (39.16)	601 (37.70)	20 (27.78)	423 (38.25)	0.79
Family history of diabetes	1749 (53.87)	165 (34.74)	876 (54.96)	41 (56.94)	667 (60.31)	<0.0001
Continuous variables, mean (SE)						
Age at enrollment (years)	46.00 (0.68)	40.59 (1.90)	44.57 (0.78)	53.96 (3.04)	49.80 (1.27)	<0.0001
Annual household income (US dollars)	22360 (872.29)	19617 (1724.54)	24563 (1284.32)	14043 (1351.98)	20561 (1084.07)	<0.0001
Years of education	10.41 (0.15)	11.58 (0.39)	10.71 (0.17)	10.24 (0.69)	9.56 (0.25)	<0.0001
MET minutes/wk. of all activity	1913.02 (384.71)	1393.1 (445.87)	2384.2 (646.06)	167.15 (107.01)	1447.24 (591.89)	0.0002
MET minutes/wk. of moderate and vigorous activity	1217.83 (154.52)	1115.31 (261.88)	1572.37 (274.14)	67.47 (48.29)	768.36 (151.68)	<0.0001
Total cholesterol (mg/dL)	183.58 (1.14)	176.41 (2.62)	182.76 (1.67)	188.24 (7.55)	187.66 (1.85)	<0.0001
Triglycerides (mg/dL)	162.37 (3.62)	98.8 (3.25)	124.83 (3.51)	195.41 (13.61)	238.58 (7.11)	<0.0001
HDL cholesterol (mg/dL)	46.45 (0.38)	53.73 (1.06)	49.41 (0.44)	42.67 (1.32)	39.64 (0.45)	<0.0001
LDL cholesterol (mg/dL)	107.38 (1.00)	103.14 (2.28)	109.86 (1.49)	108.64 (6.82)	105.83 (1.72)	0.06
Body mass index (kg/m^2^)	30.99 (0.21)	22.44 (0.16)	31.56 (0.24)	22.53 (0.47)	34.08 (0.32)	<0.0001
Waist circumference (cm)	102.87 (0.47)	84.69 (0.91)	103.35 (0.52)	86.98 (0.91)	110.3 (0.67)	<0.0001
Waist-to-hip ratio	0.93 (0.002)	0.88 (0.01)	0.93 (0.003)	0.89 (0.01)	0.96 (0.003)	<0.0001
Body fat (%)	35.59 (0.47)	25.80 (0.88)	36.51 (0.65)	27.69 (3.39)	38.33 (0.76)	<0.0001
C-reactive protein (mg/L)	3.90 (1.04)	1.48 (1.08)	2.18 (1.09)	2.77 (1.27)	5.75 (1.05)	<0.0001
Systolic blood pressure (mmHg)	116.92 (0.55)	107.53 (1.38)	113.3 (0.54)	126.04 (3.38)	125.42 (0.99)	<0.0001
Diastolic blood pressure (mmHg)	71.19 (0.32)	66.24 (1.18)	70.07 (0.35)	71.89 (1.51)	74.83 (0.57)	<0.0001
Insulin (mg/dL)^3^	12.55 (1.02)	7.69 (1.06)	11.47 (1.02)	9.12 (1.14)	17.29 (1.03)	<0.0001
Fasting blood glucose (mg/dL)^3^	105.64 (1.01)	93.69 (1.01)	101.49 (1.01)	111.05 (1.06)	119.10 (1.02)	<0.0001
HOMA IR^3^	3.29 (1.02)	1.82 (1.07)	2.89 (1.02)	2.53 (1.17)	5.05 (1.03)	<0.0001
HbA1c (%)^3^	5.53 (1.01)	5.05 (1.02)	5.31 (1.01)	6.23 (1.04)	5.93 (1.02)	<0.0001

^1^LDL: low-density lipoprotein; Hb: hemoglobin; HDL: high-density lipoprotein; HOMA IR: homeostatic model assessment insulin resistance; MET: metabolic equivalent.

^2^All descriptive results and the models were adjusted for the probability of sampling using weights taking into consideration clustering effects arising from the same census block and household. Linear regression models were used for continuous variables and Rao-Scott *F* adjusted chi-square statistic for categorical variables.

^3^Geometric concentrations.

**Table 2:** Diabetes by overweight/obese type and metabolic health status.

Diabetes	Metabolically healthy		Metabolically unhealthy		*P* value
	Normal weight	Overweight/obese	Normal weight	Overweight/obese	
	(*n* = 475)	(*n* = 1594)	(*n* = 72)	(*n* = 1106)	
Primary analysis					
Frequency					
Yes [*n*, (%)]	57 (12.00)	349 (21.89)	26 (36.11)	446 (40.33)	<0.0001^1^
No [*n*, (%)]	401 (84.42)	1208 (75.78)	42 (58.33)	641 (57.96)	
Weighted OR (95% CI)					
Unadjusted model	Reference	2.30 (1.47, 3.60)	5.20 (2.41, 11.19)	6.23 (3.94, 9.85)	<0.0001^2^
Multivariable adjusted model 1^3^	Reference	2.25 (1.34, 3.79)	3.78 (1.57, 9.09)	5.39 (3.16, 9.20)	<0.0001^2^
Multivariable adjusted model 2^4^	Reference	2.14 (1.07, 4.28)	3.18 (1.02, 9.92)	5.01 (2.43, 10.34)	<0.0001^2^
Sensitivity analysis^5^					
Frequency					
Yes [*n*, (%)]	41 (9.53)	145 (12.49)	42 (35.90)	650 (42.24)	<0.0001^1^
No [*n*, (%)]	375 (87.21)	982 (84.58)	68 (58.12)	867 (56.34)	
Weighted OR (95% CI)					
Unadjusted model	Reference	1.48 (0.87, 2.54)	4.25 (1.74, 10.37)	7.81 (4.56, 13.37)	<0.0001^2^
Multivariable adjusted model 1^3^	Reference	1.47 (0.83, 2.61)	2.93 (1.04, 8.23)	6.25 (3.54, 11.02)	<0.0001^2^
Multivariable adjusted model 2^4^	Reference	1.36 (0.59, 3.13)	3.23 (1.08, 10.01)	6.57 (2.81, 15.36)	<0.0001^2^

^1^ *F* approximation of Rao-Scott design adjusted chi-square test *P* value.

^2^ *P* values from Wald chi-square test for the effect of overweight/obese phenotype.

^3^Adjusted for age at enrollment. Other covariates were not significant and not included in the final model. The models were adjusted for the probability of sampling using weights taking into consideration clustering effects arising from the same census block and household.

^4^Adjusted for age at enrollment and family history of diabetes. Restricted to the participants who had data for family history of diabetes (*n* = 2,234, 68%).

^5^The definition of metabolically health included glucose component.
