# Supplementary material for: Metabolic Health Has Greater Impact on Diabetes than Simple Overweight/Obesity in Mexican Americans
Source: J Diabetes Res. 2016 Jan 10;2016:4094876. doi: 10.1155/2016/4094876 (PMC4736910; doi:10.1155/2016/4094876)
Supplement: Supplementary file 1 — Similar patterns as in Table 1 showed that metabolically unhealthy phenotypes had significantly elevated mean values of total cholesterol, triglycerides, high density lipid cholesterol (HDLC), fasting glucose and insulin, HOMA IR, HbA1c, CRP, and blood pressure compared with metabolically healthy phenotypes. They were more likely to be older, cigarette smokers and unemployed, less well educated, and less likely to meet the recommended guidelines for physical activity of more than 600 MET-minutes/week. They had lower household income but more frequent family history of diabetes (all Ps < 0.05). [file 4094876.f1.pdf]

Supplemental Table 1. Cohort Demographics and Metabolic Characteristics Stratified by Overweight and Obese Types and Metabolic Health Status: Cameron County Health Cohort Study (2003-2014)[1,2]

Variable	Total (n=3247)	Metabolically Healthy			Metabolically Unhealthy			*P*-value
		**Normal weight** (n=475, 14.63%)	**Overweight** (n=726, 22.36%)	**Obese** (n=868, 38.82%)	**Normal weight** (n=72, 2.22%)	**Overweight** (n=314, 9.68%)	**Obese** (n=792, 24.39%)	
**Categorical variables, n (%)**								
Men	1108 (34.11)	139 (29.26)	284 (39.12)	260 (29.95)	25 (34.72)	120 (38.22)	280 (35.35)	0.0004
Employed	1618 (49.83)	242 (50.95)	391 (53.86)	431 (49.65)	25 (34.72)	146 (46.50)	383 (48.36)	0.005
Education, below high school	1693 (52.14)	195 (41.05)	372 (51.24)	441 (50.81)	37 (51.39)	188 (59.87)	460 (58.08)	<0.0001
Met minimum recommendations for physical activity of ≥ 600 MET-minutes/week	337 (10.38)	48 (10.11)	101 (13.91)	91 (10.48)	1 (1.39)	34 (10.83)	62 (7.83)	0.01
Met recommendations of ≥ 5 servings of fruit & vegetables per day	113 (3.48)	9 (1.89)	35 (4.82)	32 (3.69)	4 (5.56)	11 (3.50)	22 (2.78)	0.06
Current smokers	495 (15.24)	64 (13.47)	100 (13.77)	122 (14.06)	14 (19.44)	60 (19.11)	135 (17.05)	0.03
Ever smokers	984 (30.3)	119 (25.05)	220 (29.99)	258 (29.72)	21 (29.17)	111 (35.35)	255 (32.20)	0.04
Ever alcohol drinkers	1230 (37.88)	186 (39.16)	293 (40.36)	308 (35.48)	20 (27.78)	120 (38.22)	303 (38.26)	0.13
Family history of diabetes	1749 (53.87)	165 (34.74)	354 (48.76)	522 (60.14)	41 (56.94)	175 (55.73)	492 (62.12)	<0.0001
**Continuous variables, Mean (SE)**								
Age at enrollment (years)	46.00 (0.68)	40.59 (1.90)	43.59 (1.26)	45.48 (0.92)	53.96 (3.04)	52.42 (1.89)	48.81 (1.35)	<.0001
Annual household income (US dollars)	22360 (872.29)	19617 (1725.45)	24646 (1830.93)	24483 (1931.39)	14043 (1352.69)	18623 (1657.21)	21207 (1318.70)	<.0001
Years of education	10.41 (0.15)	11.58 (0.39)	11.04 (0.24)	10.40 (0.24)	10.24 (0.69)	9.44 (0.33)	9.60 (0.34)	<.0001
MET minutes/wk. of all activity	1913.02 (384.71)	1393.1 (447.28)	2124.99 (767.66)	2600.89 (1076.99)	167.15 (107.35)	891.94 (456.73)	1667.76 (797.13)	0.0006
MET minutes/wk. of moderate and vigorous activity	1217.83 (154.52)	1115.31 (262.12)	1858.89 (432.87)	1289.06 (343.69)	67.47 (48.33)	707.27 (150.99)	792.63 (196.32)	<.0001
Total cholesterol (mg/dL)	183.58 (1.14)	176.41 (2.62)	182.28 (2.45)	183.21 (2.20)	188.24 (7.55)	194.44 (3.40)	185.08 (2.05)	0.002
Triglycerides (mg/dL)	162.37 (3.62)	98.80 (3.25)	119.91 (4.81)	129.41 (5.14)	195.41 (13.61)	243.70 (14.62)	236.63 (8.12)	<.0001
HDL-cholesterol (mg/dL)	46.45 (0.38)	53.73 (1.06)	50.76 (0.64)	48.15 (0.60)	42.67 (1.32)	39.79 (0.70)	39.58 (0.56)	<.0001

LDL-cholesterol (mg/dL)	107.38 (1.00)	103.14 (2.28)	109.73 (2.29)	109.98 (1.79)	108.64 (6.82)	111.11 (3.19)	103.78 (1.93)	0.04
Body mass index (kg/m^2)	30.99 (0.21)	22.44 (0.16)	27.37 (0.08)	35.45 (0.30)	22.53 (0.47)	27.81 (0.15)	36.46 (0.33)	<.0001
Waist circumference (cm)	102.87 (0.47)	84.69 (0.91)	94.73 (0.40)	111.37 (0.66)	86.98 (0.91)	98.63 (0.69)	114.73 (0.75)	<.0001
Waist-to-hip ratio	0.93 (0.002)	0.88 (0.01)	0.91 (0.005)	0.94 (0.003)	0.89 (0.01)	0.95 (0.007)	0.96 (0.003)	<.0001
Body fat (%)	35.59 (0.47)	25.80 (0.88)	31.56 (0.75)	41.11 (0.91)	27.69 (3.39)	32.98 (1.26)	40.37 (0.92)	<.0001
C-reactive protein (mg/L)	3.90 (1.04)	1.53 (1.08)	2.02 (1.06)	3.28 (1.05)	1.86 (1.15)	2.86 (1.09)	3.29 (1.07)	<.0001
Systolic blood pressure (mmHg)	116.92 (0.55)	107.53 (1.38)	112.06 (0.77)	114.47 (0.74)	126.04 (3.38)	126.24 (1.50)	125.11 (1.19)	<.0001
Diastolic blood pressure (mmHg)	71.19 (0.32)	66.24 (1.18)	68.83 (0.48)	71.23 (0.50)	71.89 (1.51)	73.79 (0.91)	75.22 (0.69)	<.0001
Insulin (mg/dL)[3]	12.55 (1.02)	7.70 (1.07)	9.51 (1.04)	13.75 (1.03)	9.12 (1.14)	12.31 (1.05)	19.69 (1.03)	<.0001
Fasting blood glucose (mg/dL)[3]	105.64 (1.01)	93.76 (1.01)	99.32 (1.01)	103.31 (1.01)	110.92 (1.07)	111.70 (1.03)	121.86 (1.02)	<.0001
HOMA IR[3]	3.29 (1.02)	1.82 (1.07)	2.33 (1.04)	3.50 (1.03)	2.53 (1.17)	3.39 (1.05)	5.90 (1.04)	<.0001
HbA1c (%)[3]	5.53 (1.01)	5.04 (1.02)	5.20 (1.02)	5.43 (1.01)	6.25 (1.04)	5.69 (1.03)	6.04 (1.02)	<.0001

[1]Abbreviation: LDL: low-density lipoprotein; Hb: hemoglobin; HDL: high-density lipoprotein; HOMA IR: homeostatic model assessment insulin resistance; MET: metabolic equivalent

[2]All descriptive results and the models were adjusted for the probability of sampling using weights taking into consideration clustering effects arising from the same census block and household. Linear regression models were used for continuous variables and Rao-Scott F adjusted chi-square statistic for categorical variables.

[3] Geometric concentrations.

Supplemental Table 2 Diabetes by Overweight and Obese Type and Metabolic Health Status

**Diabetes**	**Metabolically Healthy**			**Metabolically Unhealthy**			***P*-value**
	**Normal weight** (n=475)	**Overweight (n=726)**	**Obese (n=868)**	**Normal weight (n=72)**	**Overweight (n=314)**	**Obese (n=792)**	
Frequency							
Yes [n, (%)]	57 (12.00)	129 (17.77)	220 (25.35)	26 (36.11)	97 (30.89)	349 (44.07)	<.0001[1]
No [n, (%)]	401 (84.42)	578 (79.61)	630 (72.58)	42 (58.33)	206 (65.61)	435 (54.92)	
Weighted OR (95% CI)							
Unadjusted model	Reference	1.77 (1.06, 2.94)	2.85 (1.79, 4.52)	5.20 (2.41, 11.19)	4.21 (2.31,7.69)	7.16 (4.40, 11.66)	<.0001[2]
Multivariable-adjusted model 1 [3]	Reference	1.76 (0.98, 3.18)	2.75 (1.61, 4.68)	3.76 (1.56, 9.11)	3.25 (1.71,6.19)	6.50 (3.65, 11.55)	<.0001[2]
Multivariable-adjusted model 2 [4]	Reference	1.69 (0.76, 3.74)	2.60 (1.31, 5.18)	3.17 (1.01, 9.99)	3.50 (1.53,7.99)	5.66 (2.60, 12.31)	<.0001[2]

[1] F approximation of Rao-Scott design-adjusted Chi-square test *P*-value.
[2] *P*-values from Wald Chi-square test for the effect of overweight/obese phenotype.
[3] Adjusted for age at enrollment. Other covariates were not significant and not included in the final model. The models were adjusted for the probability of sampling using weights taking into consideration clustering effects arising from the same census block and household.
[4] Adjusted for age at enrollment and family history of diabetes. Restricted to the participants who had data for family history of diabetes (n=2,234, 68%).
